# Research on Time Difference Prediction of RTD Fluxgate Sensors Based on an Improved Transformer Neural Network

**DOI:** 10.3390/s26154776

**Published:** 2026-07-27

**Authors:** Guo Li, Na Pang, Haibo Guo, Yuhan Yang, Xu Hu

**Affiliations:** College of Computer Science and Technology, Beihua University, No. 3999 East Binjiang Road, Jilin 132013, China; lg@beihua.edu.cn (G.L.); ghb@beihua.edu.cn (H.G.); yangyuhan510@sina.com (Y.Y.); hx@beihua.edu.cn (X.H.)

**Keywords:** RTD-fluxgate, Transformer model, time difference, data prediction

## Abstract

Accurately predicting time-difference signals is a key prerequisite for extracting effective temporal characteristics from the output of RTD fluxgate sensors and enhancing the measurement reliability of the sensors. However, the nonlinear characteristics and temporal dependencies of residence time difference (RTD)-fluxgate time-difference signals make accurate prediction difficult. In this study, an improved Transformer neural network model is proposed for RTD-fluxgate time-difference signals. By introducing positional encoding, the proposed method incorporates temporal position information into the Transformer model, enabling effective extraction of temporal features from sensor output signals. To alleviate overfitting during model training, dropout and weight decay strategies are introduced to improve the generalization capability of the prediction model. The predicted time-difference signals are analyzed and the key temporal features are extracted for sensor signal processing applications. The proposed method is compared with feedforward neural networks and long short-term memory networks. Experimental results obtained under the same constant magnetic field demonstrate that the proposed method improves cosine similarity (CS) by 37% and 4%, and reduces mean square error (MSE) by 29% and 2%, respectively. The results verify the effectiveness of the proposed approach for RTD-fluxgate time-difference signal prediction and provide a data-driven method for sensor signal analysis and performance evaluation in magnetic measurement applications. This method provides a novel approach for predicting the time-difference signals of RTD fluxgate sensors, offering a reliable reference for subsequent signal processing and improving the accuracy and consistency of magnetic field data.

## 1. Introduction

Unmanned aerial vehicle (UAV) geomagnetic surveying, as an emerging magnetic measurement technology, has attracted increasing attention in geological mapping, resource exploration, and military detection due to its advantages of flexibility, low cost, and wide-area measurement capability. Compared with traditional magnetic measurement platforms, UAV-based magnetic sensing systems require sensors with compact structure, low power consumption, high sensitivity, and strong environmental adaptability. The residence time difference (RTD)-fluxgate sensor determines the external magnetic field by measuring the time difference between the positive and negative saturation intervals of the magnetic core under alternating excitation. Compared with conventional even-harmonic fluxgate sensors based on harmonic amplitude detection, the RTD-fluxgate sensor reduces the influence of harmonic interference and avoids strict structural symmetry requirements. Therefore, it provides advantages such as simple detection processes, strong anti-interference capability, miniaturization potential, and digital implementation, making it suitable for UAV geomagnetic measurement applications [1,2,3,4].

However, in practical measurements, RTD fluxgate sensors are inevitably affected by environmental interference, electromagnetic disturbances, and the sensor’s own noise. These factors cause fluctuations in the measured time-difference signals, reduce measurement stability, and increase the complexity of subsequent signal analysis. Therefore, accurately modeling and predicting the temporal characteristics of these signals is of great significance for evaluating sensor performance and improving measurement reliability.

Traditional methods, such as feedforward neural networks (FNNs) and long short-term memory (LSTM) networks, have been applied to time-series prediction tasks. However, FNNs have limited capability in extracting temporal dependencies, while LSTM networks rely on sequential calculations, resulting in increased computational complexity when processing long sequences. Moreover, error accumulation during long-term prediction may reduce prediction accuracy and affect subsequent signal processing performance [5,6,7,8].

Recently, the Transformer neural network model has achieved excellent performance in sequence modeling by introducing the self-attention mechanism. Compared with traditional recurrent neural networks, the Transformer neural network model can effectively capture long-range dependencies between different positions in a sequence and provides efficient parallel computation capabilities, making it suitable for processing sensor time-series signals [9,10,11]. However, the original Transformer architecture lacks inherent sequential information, which limits its ability to directly characterize the temporal order of RTD-fluxgate time-difference signals [12,13,14].

To address the above issues, this paper proposes an improved Transformer-based prediction method for RTD fluxgate time-difference signals. A positional encoding mechanism is introduced into the Transformer architecture to explicitly embed temporal position information into the model, thereby enhancing its capability to capture the sequential order and temporal dependencies of the signals. Meanwhile, dropout and weight decay regularization strategies are incorporated during training to reduce model complexity and mitigate overfitting. The improved model can effectively capture long-range temporal dependencies and local fluctuation patterns of the signals, leading to higher prediction accuracy. The proposed method provides strong support for high-precision prediction of RTD fluxgate time-difference signals and can be applied to sensor signal analysis and processing in UAV-based geomagnetic survey applications.

## 2. The Working Principle of the RTD-Type Fluxgate Sensor

**Figure 1:**
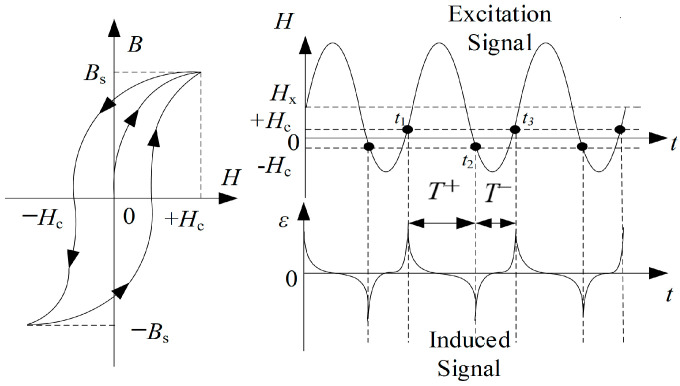
Voltage Pulse Output from the Induction Coil.

The RTD-fluxgate sensor utilizes soft magnetic materials with high magnetic permeability as the sensitive element core. When an external magnetic field $H_{x}$ is present, it affects the time the core remains in positive and negative saturation states. The measurement of the magnetic field $H_{x}$ is achieved by detecting the time difference between these saturation states. The sensor’s sensitive element core hysteresis loop and the output of the induced voltage pulse are shown in Figure 1. In practical measurements, the time intervals $T^{+}$ and $T^{-}$ between the peaks of the induced voltage pulses are detected to obtain the time difference ΔT between the two stable states, thereby measuring the magnetic field $H_{x}$ [15,16,17].

Within a cycle, when the core reaches saturation, the peak moment of the induced voltage pulse occurs at $t_{i}$ (*i* = 1, 2, 3). The relationship between the peak moments *t*_1_, *t*_2_, the excitation magnetic field $H_{e}(t)$, the measured magnetic field $H_{x}$, and the coercive force of the core ±$H_{c}$ can be derived from the magnetic core hysteresis characteristics. Accordingly, the relationship between the excitation period $T_{e}$, the excitation magnetic field $H_{e}(t)$, the measured magnetic field $H_{x}$, and the coercive force of the core ±$H_{c}$ is as follows:

(1) $$\{\begin{array}{c} t_{1}:H_{x}+H_{e}(t_{1})=H_{c}\\ t_{2}:H_{x}+H_{e}(t_{2})=-H_{c}\\ t_{3}:t_{3}=t_{1}+T_{e} \end{array}\Rightarrow \{\begin{array}{c} t_{1}=H_{e}^{-1}(H_{c}-H_{x})\\ t_{2}=H_{e}^{-1}(-H_{c}-H_{x})\\ t_{3}=t_{1}+T_{e} \end{array}$$

As shown in Figure 1, the time interval between the peak values of the induced voltage pulses is expressed as follows:

(2) $$\;\;\;\;\;\{\begin{array}{c} T^{+}=t_{2}-t_{1}\\ T^{-}=t_{3}-t_{2}\\ T_{e}=T^{+}+T^{-} \end{array}$$

Combining Equations (1) and (2), the relationship between ΔT and $H_{x}$ is derived as follows:

(3) $$\begin{array}{cc} \Delta T & =T^{+}-T^{-}\\ & =2\left[H_{e}^{-1}\left(-H_{c}-H_{x}\right)-H_{e}^{-1}\left(H_{c}-H_{x}\right)\right]-T_{e} \end{array}$$

When a trapezoidal wave magnetic field is used for excitation, assuming that the total time of the inclined sides within a cycle is *t*′, and the stable state time is *t*″, with *T_e_* being the excitation period, then *t*′ + *t*″ = *T_e_*. Where *N* denotes the cycle index of the excitation signal the slope of the inclined sides of the trapezoidal wave is *±a*, and the stable amplitude is *±H_m_*, expressed as follows:

(4) $$\;H_{e}\left(t\right)=\;\;\{\begin{array}{c} \begin{array}{cc} at\;\;\;\; & NT_{e}-\frac{t^{\prime }}{4}<t<NT_{e}+\frac{t^{\prime }}{4}\\ H_{m}\;\;\;\; & NT_{e}+\frac{t^{\prime }}{4}<t<NT_{e}+\frac{T_{e}}{2}-\frac{t^{\prime }}{4}\\ -at\;\;\;\; & NT_{e}+\frac{T_{e}}{2}-\frac{t^{\prime }}{4}<t<NT_{e}+\frac{T_{e}}{2}+\frac{t^{\prime }}{4}\\ -H_{m}\;\;\;\; & NT_{e}+\frac{T_{e}}{2}+\frac{t^{\prime }}{4}<t<NT_{e}+T_{e}-\frac{t^{\prime }}{4} \end{array} \end{array}$$

**Figure 2:**
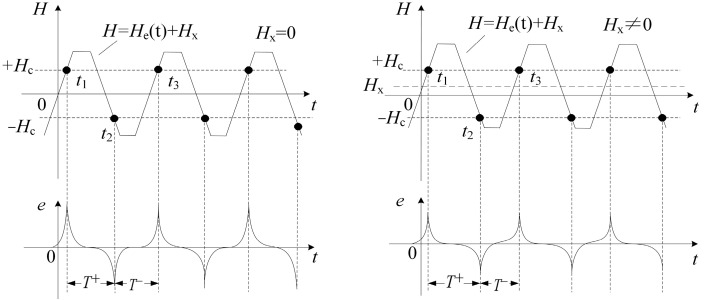
The Relationship Between Magnetic Fields When the Core is Saturated with Trapezoidal Wave Excitation.

When the total magnetic field strength *H* acting on the sensitive element core reaches the saturation state of the core, the relationship between the excitation magnetic field $H_{e}(t)$, the measured magnetic field $H_{x}$, and the coercive force $H_{c}$ is as shown in Figure 2.

As shown in Figure 2, when the total magnetic field strength reaches the saturation state of the core, i.e., when the total magnetic field strength reaches the coercive forces *t*_1_, *t*_2_, and *t*_3_, the moments of positive and negative saturation of the core +*H_c_* and −*H_c_*, respectively. Under this condition, substituting Equation (4) into Equation (1), the moments *t*_1_, *t*_2_, and *t*_3_ satisfy:

(5) $$\{\begin{array}{c} t_{1}:H_{x}+at_{1}=H_{c}\\ t_{2}:H_{x}-a(t_{2}-\frac{T_{e}}{2})=-H_{c}\\ t_{3}:t_{3}=t_{1}+T_{e} \end{array}\Rightarrow \{\begin{array}{c} t_{1}=\frac{H_{c}-H_{x}}{a}\\ t_{2}=\frac{H_{c}+H_{x}}{a}+\frac{T_{e}}{2}\\ t_{3}=t_{1}+T_{e} \end{array}$$

From Equation (5), the positive and negative saturation time intervals $T^{+}$ and $T^{-}$ between the induced voltage pulses within one excitation period $T_{e}$, when the magnetic field strength reaches the saturation state of the core at moments *t*_1_, *t*_2_, and *t*_3_, are as shown in Equation (6):

(6) $$\{\begin{array}{c} T^{+}=t_{2}-t_{1}=\frac{2H_{x}}{a}+\frac{T_{e}}{2}\\ T^{-}=t_{3}-t_{2}=-\frac{2H_{x}}{a}+\frac{T_{e}}{2} \end{array}$$

From Equation (6), it can be seen that under trapezoidal wave magnetic field excitation, a linear relationship between the output time difference ΔT and the external magnetic field $H_{x}$ is obtained through the difference between *T*^+^ and *T*^−^, further taking the partial derivative with respect to $H_{x}$, the expression for the sensor sensitivity *S* can be derived as follows:

(7) $$\Delta T=T^{+}-T^{-}=\frac{4H_{x}}{a}=\frac{4H_{x}t^{\prime }}{4H_{m}}=\frac{H_{x}t^{\prime }}{H_{m}}$$

(8) $$\;S=\frac{\partial \Delta T}{\partial H_{x}}=\frac{4}{a}=\frac{t^{\prime }}{H_{m}}$$

From the working principle of the RTD-fluxgate sensor, it is known that before magnetic field calibration, the sensor output is a series of longtime difference sequences. Due to the influence of external noise, the time difference data fluctuates significantly. To better capture the underlying patterns in the RTD-fluxgate sensor output, it is necessary to choose an appropriate neural network model to predict the time difference data of the sensor output, providing a reference for subsequent signal processing and improving sensor accuracy [18,19]. Given the characteristic of long time series in time difference data, the Transformer model excels in capturing long-distance dependencies but is prone to overfitting, which reduces the model’s generalizability and prediction accuracy. Therefore, it is necessary to improve the Transformer model to prevent overfitting and enhance the accuracy of time difference data training [20,21,22,23].

## 3. Research on Time Difference Prediction Using Improved Transformer Model

### 3.1. Transformer Model Prediction Research

The Transformer model, based on the Self-Attention mechanism, is widely used in natural language processing and time-series data modeling. Compared to traditional Recurrent Neural Networks (RNN), the Transformer consists of two components: the Encoder and the Decoder. When using the Transformer model for predicting the time difference in fluxgate sensor outputs, the Self-Attention mechanism captures long-range dependencies, improving prediction accuracy. Additionally, its strong global information-capturing ability overcomes the gradient issues inherent in traditional neural networks, while its parallel processing capability enhances processing efficiency [24,25,26].

**Figure 3:**
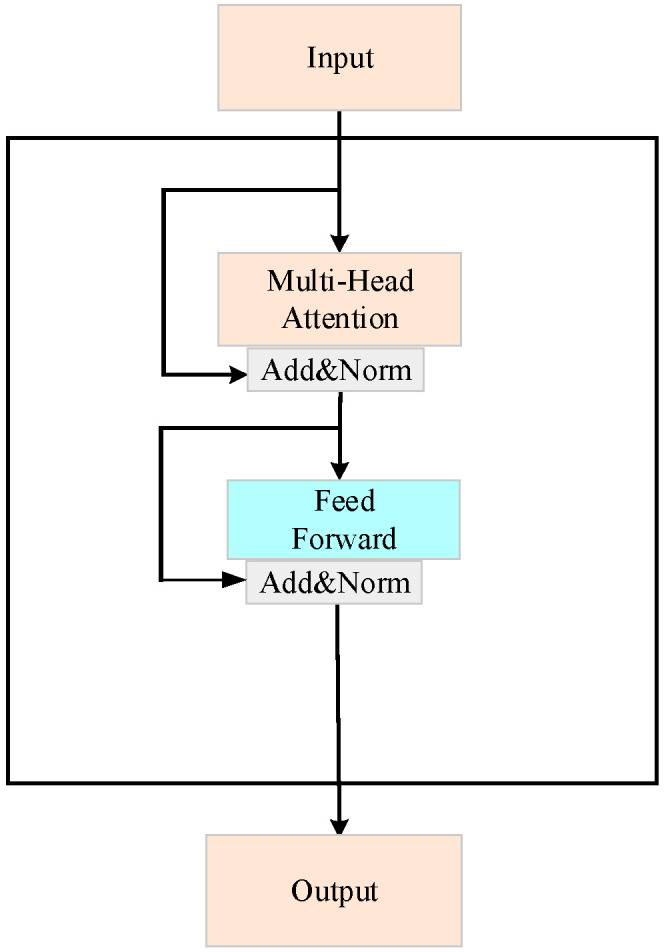
Transformer Encoder Structure Diagram.

The basic structure of the Encoder part in the Transformer is shown in Figure 3. This part consists of a Multi-Head Self Attention (MHSA) module and a Feedforward Neural Network (FNN). The principle expressions of the multi-head self-attention module are shown in Equations (9) and (10):

(9) $$\{\begin{array}{c} Q=W^{Q}I\\ K=W^{K}I\\ V=W^{V}I \end{array}$$

(10) $$\mathrm{Attention}(Q\cdot K\cdot V)=\mathrm{softmax}(\frac{QK^{T}}{\sqrt{d_{k}}}V)$$

Herein, Q, K, and V represent the matrices for Query, Key, and Value, respectively. These three values are obtained by multiplying the input I of the network nodes with three different matrices $W^{Q}$, $W^{K}$, and $W^{V}$. The Query matrix and the Key matrix compute attention weights through dot product to measure the correlations between different positions in the sequence, while the Value matrix aggregates information based on these weights. $\sqrt{d_{k}}$ is the scaling factor, which is the variance of the matrix obtained after performing the inner product operation between the Query and Key matrices. The scaling factor serves to prevent the dot product results from entering regions of extremely small gradients, thereby ensuring the stability of the training process. To enhance the training efficiency of the network, the multi-head self-attention module employs the scaled dot-product method to implement the self-attention mechanism.

In the Encoder part, the Multi-head Attention mechanism is included, which primarily projects Q, K, and V through h different linear transformations and performs calculations. The different Attentions are then concatenated, as shown in Equation (11), which can improve the computational efficiency of the model.

(11) $$MultiHead(Q,K,V)=Concat(head_{1},\ldots ,head_{h}),\;\mathrm{where}\;{\mathrm{head}}_{i}=Attention(QW_{i}^{Q},KW_{i}^{K},VW_{i}^{V})$$

Each attention head learns different types of dependencies in the input sequence by performing attention computations in distinct subspaces. The Self-Attention layer can establish correlations between different positions, effectively capturing long-range dependencies in the input sequence, which is crucial for the long-time series training of the RTD-fluxgate sensor.

### 3.2. Construction of Time Difference Prediction Model Based on Transformer

The positional encoding matrix $P=R^{n\cdot l\cdot d}$ and the input encoding matrix $I=R^{n\cdot l\cdot d}$, where n is the number of time periods, l is the maximum number of observation points in a time period, and d is the dimension of the embedding vector, make up the encoder’s input in the Transformer model. Although P and I share the same tensor shape, they represent different types of information. Specifically, P is the positional encoding, which explicitly injects temporal order information into the model, thereby complementing the sequence position representation; I is the embedded representation of the input signal, used to encode the amplitude information and local features of the RTD-fluxgate time-series signal. The RTD-fluxgate sensor produces time-series signals containing time-difference variations, in which the positional relationship between samples is highly correlated with the signal dynamics [27]. Therefore, positional information is critical for subsequent modeling tasks. However, the self-attention mechanism primarily computes attention weights based on similarity between samples and does not inherently impose sequential order constraints, meaning it cannot directly capture the temporal structure of the RTD-fluxgate signal. The time-difference signals output by the RTD-fluxgate sensor possess strict temporal physical significance different sampling moments correspond to different saturation states of the magnetic core under alternating excitation, and the temporal sequence itself carries critical information about magnetic field variations. To address this limitation, positional encoding is introduced at the input stage of the Transformer model, enabling the model to preserve its global dependency modeling capability while also incorporating explicit temporal order information, thereby improving its ability to represent RTD-fluxgate time-series features.

Positional encoding is an additional numerical vector specifically designed to identify the exact position of input data within the time series, enabling the model to understand the temporal dependencies present in the data. It is typically computed using fixed sine and cosine functions to guarantee the uniqueness of encodings at different positions while allowing the Transformer model to effectively learn them. Specifically, for a time step t, the positional encoding vector is computed as follows:

(12) $$\;\;\;PE_{\left(t,2i\right)}=\sin\left(\frac{t}{{10,000}^{\frac{2i}{d}}}\right),\;PE_{\left(t,2i+1\right)}=cos\left(\frac{t}{{10,000}^{2i/d}}\right)$$

Here, *t* denotes the current time step’s position, *d* refers to the encoding vector’s dimension, and *i* indicates the index within the positional encoding vector. This formulation generates a unique encoding for each position using sine and cosine functions. This positional encoding method is derived from the original Transformer model design, providing explicit temporal position information support for sequence modeling [28]. By incorporating the positional encoding matrix, the Transformer model can effectively capture dependencies between any two points in the time series, recognize and leverage long-term dependency patterns in the sequence, and thus better adapt to the time difference sequences of the RTD-fluxgate sensor.

Overfitting is a frequent problem because RTD-fluxgate time-difference signals exhibit large fluctuations and long sequence characteristics. In sensor data prediction tasks, the time-difference sequences are long and exhibit large numerical fluctuations, making model weights prone to excessive growth during training, which leads to oversensitivity to minor variations in the input. We fix this by adding weight decay to the optimizer. In particular, weight decay reduces the complexity of the model and lessens the chance of overfitting by regularizing the model’s weights throughout the optimization process. The following is the expression:

(13) $$C=\frac{1}{2}\lambda (\sum_{i=1}^{L}||W^{i}|{|}_{F}^{2})$$

Here, *λ* is the coefficient controlling the regularization strength, *L* denotes the number of weight matrices included in the regularization term, $W^{i}$ represents the i-th weight matrix, and $||\ldots |{|}_{F}$ denotes the Frobenius norm. This form represents a standard L2 regularization approach, which reduces model complexity by constraining the magnitude of model parameters, thereby enhancing the model’s generalization capability. By adjusting the optimizer’s parameters, the weight decay term can be tuned, thereby achieving regularization of the model’s weights [29,30,31].

In addition, when training deep neural networks, randomly dropping out some neurons can reduce overfitting and enhance the model’s ability to generalize. This technique is known as Dropout [18,22]. The RTD-fluxgate sensor is affected by noise during practical measurements, causing the model to easily over-rely on certain local features or even directly fit the noise during training. Therefore, to enhance the model’s robustness and prevent overfitting, we incorporate Dropout into the Self-Attention layer of the Transformer model. By randomly deactivating different subsets of neurons during training, Dropout enhances the model’s robustness and enables the model to learn diverse feature representations, which helps better represent RTD-fluxgate time-difference signals. Dropout approximates a form of Bayesian learning, with y=f(x;θ) representing the neural network to be learned. From a theoretical perspective, Dropout randomly deactivates some neurons during training to achieve an implicit ensemble of multiple sub-networks, thereby approximating a model averaging mechanism. Assuming that the parameter θ is a random vector with a prior distribution q(θ), the Bayesian prediction formula is as shown in Equation (14).

(14) $$\begin{array}{c} E_{q(\theta )}[y]=\int_{q}\;f(x;\theta )q(\theta )d\theta \approx \frac{1}{M}\sum_{m=1}^{M}\;f(x;\theta_{m}) \end{array}$$

In the formula, $y=f(x;\theta_{m})$ represents the network after the m-th application of dropout, with $\theta_{m}$ being a single sample of all parameters θ, and *M* represents the overall count of dropout operations. This expression reveals the regularization mechanism of Dropout, which enhances the model’s generalization capability through implicit averaging over multiple sub-networks.

**Figure 4:**
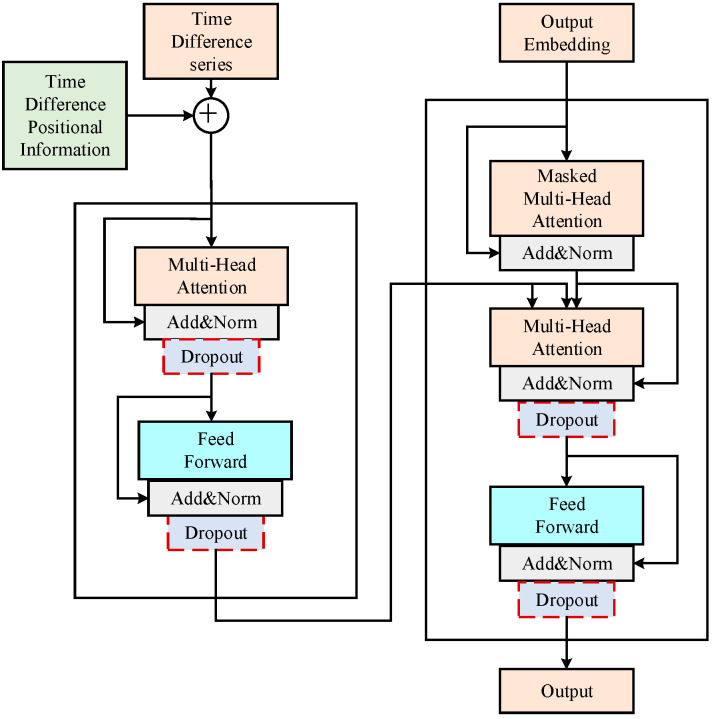
Architecture Diagram of the Improved Transformer Model.

As previously discussed, both weight decay and dropout serve as effective strategies to mitigate model overfitting. In this study, these two techniques are integrated to strike a balance between model complexity and generalization capability. In the context of time difference data generated by the RTD-fluxgate sensor, combining weight decay and dropout with positional encoding enhances temporal feature representation, improves generalization performance, and reduces the likelihood of overfitting. Since weight decay is a global setting for the entire model, it is added to the global environment when defining the model. Dropout is applied following both the Multi-Head Attention mechanism and the Feedforward Neural Network. The positional encoding of the time difference data is input into the Encoder module along with the time difference data. The improved Transformer model architecture is shown in Figure 4.

## 4. Simulation Experiments and Analysis

In this section, we evaluate and compare the prediction performance of FNN, LSTM, and an improved Transformer on time-difference sequences. A model that can more accurately predict values close to the true ones demonstrates a better understanding of the underlying patterns in the time-difference signals, and its prediction results can provide more reliable data references for subsequent signal processing. Therefore, this study adopts “prediction accuracy” as the quantitative criterion for evaluating model prediction performance. In similarity evaluation, Cosine Similarity (CS) is a metric used to measure the degree of similarity between two vectors in the vector space, and is commonly applied to evaluate the consistency between predicted and actual sequences. In the context of predicting time-difference data from RTD-fluxgate sensors, CS quantifies how closely the predicted results align with the actual values in terms of both trend and shape. The formula for CS is given as follows:

(15) $$CS=\frac{\sum_{i=1}^{N}\;y_{i}{\hat{y}}_{i}}{\sqrt{\sum_{i=1}^{N}\;y_{i}^{2}}\cdot \sqrt{\sum_{i=1}^{N}\;{\hat{y}}_{i}^{2}}}$$

where $y_{i}$ denotes the ground-truth value, ${\hat{y}}_{i}$ denotes the predicted value, and *N* is the number of samples. A higher CS value, approaching 1, indicates that the predicted sequence closely matches the ground truth in directional variation.

Mean Squared Error (MSE), on the other hand, measures the average squared difference between the predicted and actual values, reflecting the magnitude of prediction deviation. The formula for MSE is given as follows:

(16) $$MSE=\frac{1}{N}\sum_{i=1}^{N}\;(y_{i}-{\hat{y}}_{i}{)}^{2}$$

where $y_{i}$ denotes the ground-truth value, ${\hat{y}}_{i}$ denotes the predicted value, and N is the number of samples. A lower MSE indicates higher prediction accuracy and smaller residual errors.

To summarize, CS evaluates the quality of prediction from the perspective of sequence similarity, while MSE assesses it from the perspective of numerical deviation. The two metrics complement each other and are therefore jointly employed to comprehensively evaluate the training performance of different models.

The improved Transformer model was used to train on the normalized time-difference data from the RTD-fluxgate sensor in order to assess the effectiveness of the suggested technique. 1000 RTD fluxgate sensor output time-difference data points with a mean of 800 and a variance of 10 were used in MATLAB R2023a simulations. Training accounted for 80% of the simulated data, while testing accounted for 20%. With a batch size of 100, the enhanced Transformer model received training across 100 epochs. It has four attention heads, two encoder layers, and twelve hidden layers. The dropout rate was fixed at 0.05, while the weight decay parameter, λ, was set to 0.001. Next, this model’s performance was contrasted with that of LSTM and FNN.

**Figure 5:**
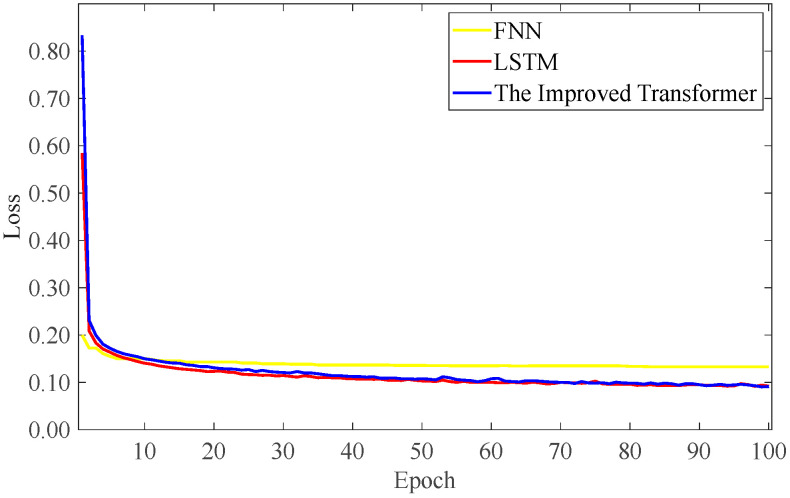
Loss Function Variation Curves of the Three Models.

The improved Transformer was trained using time-difference samples, and predictions were generated accordingly. Key metrics including fluctuations, variance, mean, MSE, and CS were evaluated for the time-difference values both prior to and following training. The comparative results are presented in Table 1. According to the table, the improved Transformer model achieved an MSE of 0.0908 and a CS of 0.867. Compared to the FNN training results, the MSE decreased by 29%, and the CS increased by 37%. Compared to the LSTM training results, the MSE decreased by 2%, and the CS increased by 4%. In terms of fluctuations, variance, and mean, the improved Transformer model generally aligned with the simulated data. These results indicate that the improved Transformer model outperforms FNN and LSTM in training time difference data. Figure 5 presents the loss functions obtained from the three training approaches. With the growth in training epochs, the loss values of all three methods gradually decrease and eventually converge. Among them, the improved Transformer model achieves the lowest converged loss, demonstrating its superior training performance.

**Figure 6:**
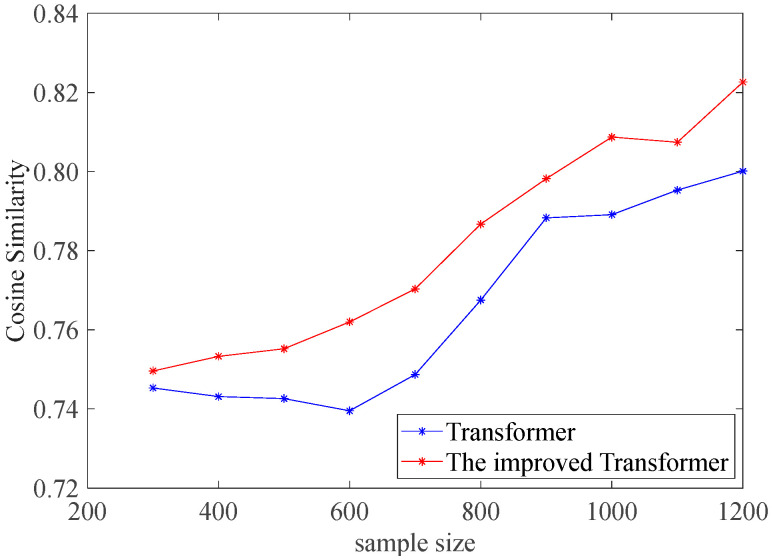
Results of the Ablation Experiment.

To further investigate the impact of different time-difference sequence lengths on model prediction performance and evaluate the effectiveness of the improved model, an comparative experiment was conducted. Ten groups of time-difference sequences with lengths ranging from 300 to 1200 were selected for prediction, and CS was adopted as the evaluation metric. Figure 6 illustrates the results, with the time-difference sequence length and the vertical axis representing CS. In cases of smaller data volumes, the difference in CS between the improved and the original Transformer models is not significant (with a sample length of 300, the CS of the original Transformer model was 0.7453, and the CS of the improved Transformer model was 0.7496, only a 0.5% improvement), but as the data volume increases, the gap becomes more apparent. When the time-difference sequence length reaches 1200, the improved Transformer model’s CS shows a 2.8% increase over that of the original Transformer.

**Figure 7:**
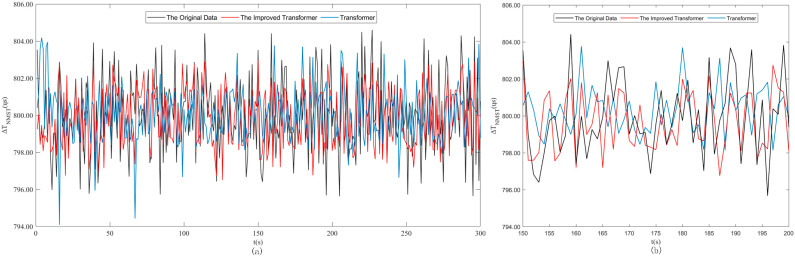
Comparison of prediction results between the Transformer and improved Transformer models: (**a**) comparison of prediction results; (**b**) magnified view of a selected interval.

Figure 7 presents a comparison between the predicted results of the improved Transformer and the original Transformer models against the simulated time-difference data. The horizontal axis corresponds to the length of the time-difference series, while the vertical axis shows the time-difference values. Figure 7a shows the comparison of prediction results, and Figure 7b provides a magnified view of a selected interval. As illustrated, the improved Transformer provides a closer match to the actual data than the original model, indicating that the proposed method can effectively improve the prediction performance of time-difference sequences.

## 5. Experiment and Preliminary Results

Due to the presence of geomagnetic fields and external magnetic interference, the tests are significantly affected. To enhance the accuracy of the laboratory tests, a Helmholtz coil is positioned in the central region of an electromagnetic shielding cylinder made of five-layer permalloy within the electromagnetic shielding room at the National Geophysical Exploration Instrument Engineering and Technology Research Center of Jilin University. The RTD-fluxgate sensor, developed by the Key Laboratory of Geophysical Exploration Equipment, Ministry of Education at Jilin University, is placed in the uniform field zone of the Helmholtz coil. A magnetic field ($H_{x}$), parallel to the axis of the sensor’s core, is applied to the sensor’s sensing unit for testing.

**Figure 8:**
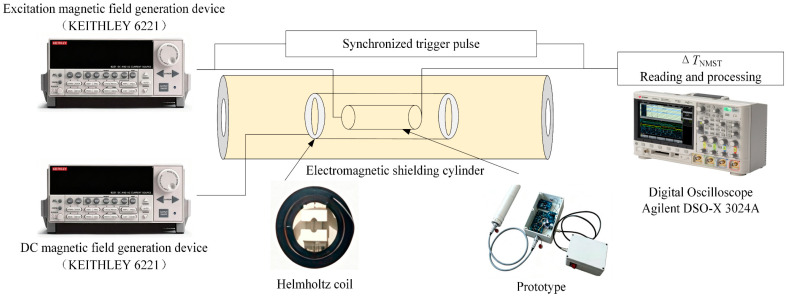
Schematic diagram of the instrument connections and experimental setup.

The Keithley 6221 precision current source is used to generate a current, which is applied across the excitation coil of the sensor to produce an excitation magnetic field. A trapezoidal waveform magnetic field is used for excitation. When the frequency is *f* = 30 Hz and the current is *I* = 60 mA, the FPGA counts the time difference using two-channel signals. The counting frequency of the FPGA is $f_{c}$ = 100 MHz. The number of time points (N) is converted into the time difference $\;(\Delta T_{\mathrm{NMST}}$) and transmitted to the STM32 for storage. The instrument connections among the components are shown in Figure 8.

**Figure 9:**
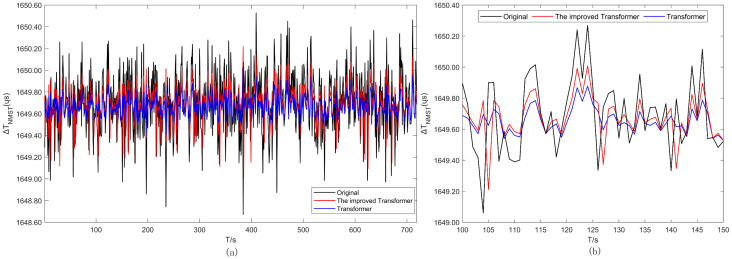
Comparison of data trained by different methods: (**a**) comparison of prediction results; (**b**) magnified view of a selected interval.

To validate the effectiveness of the proposed method, when $H_{x}$ = +50,000 nT and the observation time *t* = 1 h, the data was divided into 30 groups, and the average was taken to reduce the impact of random noise. The sensor output $\Delta T_{\mathrm{NMST}}$ was processed without any treatment, and then trained for 100 epochs using FNN, LSTM, Transformer, and the improved Transformer model (where the first 80% of the data was used for training, and the remaining 20% for prediction. The model parameters included a batch size of 100, 12 hidden layers, 2 encoder layers, and 4 attention heads). A comparison of the $\Delta T_{\mathrm{NMST}}$ values processed by different methods is shown in Figure 9. Figure 9 presents the comparison of time-difference data trained by different methods, where Figure 9a shows the comparison of prediction results, and Figure 9b provides a magnified view of a selected interval to more clearly illustrate the detailed differences in the prediction results of each model. From Figure 9, it can be observed that the prediction trend of the improved Transformer model is closer to the measured data. Compared with the original data, the improved Transformer reduced the fluctuation of the time-difference signal by 0.64 μs, decreased the variance by 0.01 μs^2^, while the mean value changed only slightly (within 0.01 μs). These results indicate that the predicted signal maintains good stability and is closer to the measured data.

**Figure 10:**
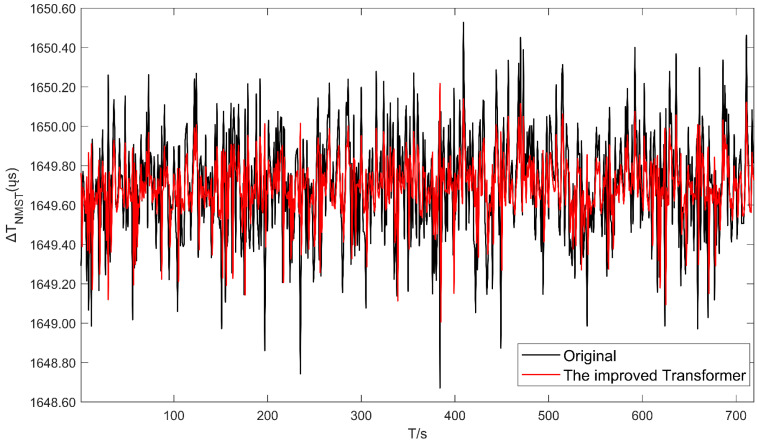
Comparison Chart of Time Difference Predictions by the Improved Transformer Model Against Actual Measurement Data.

To better compare the effectiveness of different methods for time difference prediction, a statistical comparison of the prediction results from different methods on the test set is presented in Table 2, in terms of the MSE and CS evaluation metrics. The improved Transformer model achieved an MSE of 0.0903 and a CS of 0.8496. Compared with the prediction results of the FNN, the MSE decreased by 31% and the CS increased by 34%; compared with the prediction results of the LSTM, the MSE decreased by 12% and the CS increased by 9%; and compared with the prediction results of the original Transformer model before improvement, the MSE decreased by 3% and the CS increased by 5%. Therefore, it can be concluded that the improved Transformer model outperforms the FNN, LSTM, and the original Transformer model in time-difference prediction. This performance improvement is mainly attributed to the synergistic effects of positional encoding, dropout, and weight decay. Among these, positional encoding enables the model to more accurately capture time-difference features in the time series, enhancing its ability to model temporal dependencies; dropout improves the model’s generalization capability and reduces the risk of overfitting by suppressing neuronal co-adaptation; and weight decay enhances training stability and prevents parameter oscillation by constraining the magnitude of parameter updates. Meanwhile, during the training process, the improved model exhibits a stable convergence trend, and consistent performance improvements are observed across different evaluation metrics and comparison models. These results indicate that our proposed method is reasonably stable. Figure 10 shows the comparison between the time-difference predictions of the improved Transformer model and the measured data, further validating the superiority of this model in time-difference sequence prediction. In summary, through training and prediction on measured data, it is concluded that the improved Transformer model achieves better prediction results for time difference sequences.

## 6. Conclusions and Discussion

In this paper, we proposed an improved Transformer model incorporating positional encoding, dropout, and weight decay for predicting RTD-fluxgate sensor output time-difference signals. This approach addresses the issue of missing temporal position information in the time-difference sequence, mitigates overfitting, and optimizes prediction performance. Simulation and experimental comparisons using CS and MSE as evaluation indicators reveal that, compared with FNN, LSTM, and the original Transformer, the enhanced Transformer achieves improvements of approximately 5% in CS and 3% in MSE, confirming the effectiveness of the proposed method. The results demonstrate that the improved Transformer model can effectively capture temporal dependencies in RTD-fluxgate time-difference signals. The proposed method provides a research foundation and data support for RTD-fluxgate sensor signal processing, contributing to the enhanced reliability of UAV-based aeromagnetic surveying and monitoring.

Finally, although the proposed improved Transformer model prediction method has provided a foundation for the development of UAV-based aeromagnetic surveying technology through simulation studies and experimental analysis, the experimental validation in this paper was mainly conducted under laboratory-controlled constant magnetic field conditions ($H_{x}$ = +50,000 nT) to ensure the reproducibility of the experimental results. However, the prediction performance of the actual RTD-fluxgate sensor under dynamically varying magnetic field conditions still requires further verification, and related systematic experiments will be the focus of our future work.

## Figures and Tables

**Table 1:** Statistical Comparison Table Before and After Time Difference Training.

	MSE	CS	Fluctuation (μs)	Variance (μs^2^)	Mean (μs)
simulation data			8.644	10	800
FNN	0.1293	0.6309	9.955	8.02	797.2
LSTM	0.0925	0.8317	8.036	8.76	801.2
The improved Transformer	0.0908	0.8674	8.332	9.57	799.5

**Table 2:** Statistical Comparison Table of Time Difference Data Prediction by Different Methods.

	MSE	CS	Fluctuation (μs)	Variance (μs^2^)	Mean (μs)
Original			1.8567	0.0783	1649.69
FNN	0.1323	0.6336	0.9955	0.0597	1649.59
LSTM	0.1031	0.7751	0.7392	0.0382	1649.71
Transformer	0.0932	0.8038	0.6243	0.0371	1649.66
The improved Transformer	0.0903	0.8496	1.2108	0.0639	1649.68

## Data Availability

Data are contained within the article.
